# Novel insights into water-deficit-responsive mRNAs and lncRNAs during fiber development in *Gossypium hirsutum*

**DOI:** 10.1186/s12870-021-03382-y

**Published:** 2022-01-03

**Authors:** Nan Wu, Jun Yang, Guoning Wang, Huifeng Ke, Yan Zhang, Zhengwen Liu, Zhiying Ma, Xingfen Wang

**Affiliations:** State Key Laboratory of North China Crop Improvement and Regulation, North China Key Laboratory for Crop Germplasm Resources of Education Ministry, Hebei Agricultural University, Baoding, 071001 China

**Keywords:** *Gossypium hirsutum*, Fiber, mRNA, Long noncoding RNA (lncRNA), Water deficit

## Abstract

**Background:**

The fiber yield and quality of cotton are greatly and periodically affected by water deficit. However, the molecular mechanism of the water deficit response in cotton fiber cells has not been fully elucidated.

**Results:**

In this study, water deficit caused a significant reduction in fiber length, strength, and elongation rate but a dramatic increase in micronaire value. To explore genome-wide transcriptional changes, fibers from cotton plants subjected to water deficit (WD) and normal irrigation (NI) during fiber development were analyzed by transcriptome sequencing. Analysis showed that 3427 mRNAs and 1021 long noncoding RNAs (lncRNAs) from fibers were differentially expressed between WD and NI plants. The maximum number of differentially expressed genes (DEGs) and lncRNAs (DERs) was identified in fibers at the secondary cell wall biosynthesis stage, suggesting that this is a critical period in response to water deficit. Twelve genes in cotton fiber were differentially and persistently expressed at ≥ five time points, suggesting that these genes are involved in both fiber development and the water-deficit response and could potentially be used in breeding to improve cotton resistance to drought stress. A total of 540 DEGs were predicted to be potentially regulated by DERs by analysis of coexpression and genomic colocation, accounting for approximately 15.76% of all DEGs. Four DERs, potentially acting as target mimics for microRNAs (miRNAs), indirectly regulated their corresponding DEGs in response to water deficit.

**Conclusions:**

This work provides a comprehensive transcriptome analysis of fiber cells and a set of protein-coding genes and lncRNAs implicated in the cotton response to water deficit, significantly affecting fiber quality during the fiber development stage.

**Supplementary Information:**

The online version contains supplementary material available at 10.1186/s12870-021-03382-y.

## Background

Cotton is the major source of natural fibers and the most important raw material for the textile industry. Cotton fiber development is generally defined into four distinct but overlapping stages, including fiber initiation [FI, from 2 days before anthesis to 3 ~ 6 days post anthesis (DPA)], fiber elongation (FE, primary cell wall biosynthesis, 3 ~ 20 DPA), secondary cell wall biosynthesis (SCWB, 16 ~ 40 DPA), and maturation (40 ~ 50 DPA) [1, 2]. FI, marking the start of fiber growth, is a key stage that determines cotton yield. Many genes regulating FI have been identified in cotton, such as the MYB transcription factors *GhMYB25* [3] and *GhMYB25-like* [4], protodermal factor *GbPDF1* [5], jasmonate zim-domain protein *GhJAZ2* [6], auxin efflux carrier *GhPIN3a* [7], MYB-MIXTA-like transcription factors *GhMML4* and *GhMML3* [8]. FE has been suggested as a crucial stage for determining the final fiber quality. Some genes have been characterized to play roles in this stage. For example, six *Gh14-3-3* genes are predominantly expressed at the FE stage, and overexpression of these genes promotes the longitudinal growth of fission yeast, indicating that they might participate in the regulation of fiber elongation [9]. Two transcription factors (TFs), *GhHOX3* (homoeodomain-leucine zipper TF) and *GhDEL65* (basic helix-loop-helix TF), positively regulate cotton fiber elongation [10, 11]. *GhLTPG1* (glycosylphosphatidylinositol anchored lipid transport protein) is abundantly expressed in elongating cotton fibers, and knockdown of *GhLTPG1* results in shorter fibers with the repression of FE-related gene expression [12]. Cotton fiber quality traits, including strength, micronaire, and maturity, are mostly determined at the stages of SCWB and maturation. The biology and genes involved in these two stages are much less understood and studied [13], with only MYB transcription factors reported [14, 15].

High-throughput RNA-seq technology has been used to understand complex responses and for the functional exploration of protein-coding genes. Nevertheless, it was reported that a large portion of RNAs in eukaryotes, such as humans and Arabidopsis, do not encode proteins and are known as noncoding RNAs (ncRNAs) [16, 17]. ncRNAs over 200 nucleotides in length are named long ncRNAs (lncRNAs), which are further classified as long intergenic ncRNAs (lincRNAs), natural antisense transcripts (lncNATs), long intronic ncRNAs, and lncRNAs partially overlapping with protein-coding genes [18]. Thousands of lncRNAs have been identified in several plants, expanding our understanding of the plant transcriptome. In Arabidopsis, 6510 lncRNAs were identified, among which approximately five hundred showed inducible expression patterns upon exposure to abscisic acid (ABA) and drought [19]. In *Ricinus communis*, 5356 lncRNAs were cataloged and potentially involved in regulating the development of endosperm and embryos in castor bean seeds [20]. A total of 23,651 novel lncRNAs were identified in Tibetan wild barley, of which 535 lncRNAs were differentially expressed in response to drought stress [21]. In addition, as many as 59,110, 57,944 and 40,858 actively-expressed putative lncRNAs were identified from three wheat varieties, including Kiziltan, TR39477 and TTD-22 varieties, respectively [22]. With rapid advances in whole genome sequencing analyses of cotton, including *Gossypium raimondii*, *G. arboreum*, *G. hirsutum* and *G. barbadense*, thousands of lncRNAs have been identified and shown to have potential functions in cotton growth [23, 24], fiber development (initiation and elongation) [25, 26], and various stress responses, including drought [27], salt [28], phytopathogen *Verticillium dahliae* infection [29, 30] and piercing-sucking pest *Aphis gossypii* attack [31].

Although lncRNAs have been identified in large numbers of plants and are believed to have crucial roles in development and stress responses, the functional roles and the mechanism underlying the lncRNAs is insufficient [32]. Novel functional networks will likely be defined by predicting and characterizing the interaction between lncRNAs and potential targets [33]. Emerging evidence suggests that plant lncRNAs have various mechanisms of action, mainly studied in Arabidopsis at present. LncRNA (*ASCO-lncRNA*) functions in Arabidopsis lateral root (LR) meristems by interacting with nuclear speckle RNA-binding protein (NSR), which is an alternative splicing regulator [34]. In other words, lncRNAs can hijack NSRs to affect their binding to mRNA targets. LncRNAs can bind with miRNAs by complementary sequences; thus, alterations in lncRNA abundance can modulate the action of miRNAs on downstream protein-coding genes. This mechanism of inhibition of miRNA activity was defined as target mimicry [35]. For example, lncRNA *IPS1* (induced by phosphate starvation 1) functions in regulating the phosphate starvation response in Arabidopsis by imperfect interaction with miRNA miR-399, which can guide the cleavage of *PHO2* [35, 36]. LncRNA *FRILAIR* (fruit ripening-related long intergenic RNA), a target mimic of miR397, can modulate the expression of *LAC11a* involved in strawberry fruit ripening [37]. In addition, the alteration of lncRNA expression can affect the dynamic chromatin topology, which determines the expression of neighboring genes. For instance, lincRNA APOLO transcription regulates the formation of a chromatin loop encompassing the promoter of its neighboring gene PID, a key regulator of polar auxin transport [38].

Cotton is mostly grown commercially in semiarid and arid environments. Fiber yield and quality are greatly and periodically affected by drought stress, and the severity of the problem may increase due to global climate change [39, 40]. Therefore, breeding cotton cultivars with higher yield and better fiber quality under drought conditions is becoming more urgent [41]. Some information has been available about cotton fiber development and drought resistance by the characterization of some important genes and analysis of transcriptome profiling, but the regulatory mechanism has not been absolutely elucidated to date [13, 41]. The Yellow River Basin (YRB) is one of the three major cotton production regions in China. In most years, rainfall was significantly reduced during the cotton flowering period in the YRB, which seriously affected the growth and development of cotton. To guarantee and increase cotton yield, supplementary irrigation just before flowering (SIF) is a widely used farm operation to compensate for the lack of rainfall in the YRB. However, the effect of SIFs on cotton fiber development has not been investigated in detail thus far, especially on fiber quality, and the underlying mechanism for cotton fiber cells responding to non-SIFs (water deficit). The present study presents the first research on the effects of water deficit on cotton fiber development by genome-wide analysis of mRNAs and lncRNAs in fiber cells. In particular, some bifunctional lncRNAs preferentially expressed during fiber development and involved in the water-deficit response were identified. These results will deepen our understanding of the molecular mechanism underlying fiber development under drought stress, and provide clues to accelerate the development of novel cotton cultivars with improved yield potential, fiber quality, and adaptability to drought conditions.

## Methods

### Plant material and irrigation treatments

*G. hirsutum* cv. Nongdamian 13 (ND13) was planted in a rainout shelter at the Teaching Experimental Station of Hebei Agricultural University (38°49′N, 115°26′E), Baoding, China. The rows of plants were spaced 100 cm apart and 30 cm between plants within a row. When the first white bloom was observed, the normal irrigation cotton field (NI) received irrigation with 675 m^3^/ha of water, but the water deficit field (WD) did not. All other agronomic management practices were kept normal and uniform for NI and WD. The soil drought level was determined with the soil relative water content (SRWC) as previously described [42]. The cultivar ND13 was collected from Hebei Province in China by Hebei Agricultural University and approved by Hebei Provincial Crop Variety Certification Committee with an accession number G10072. All necessary permissions for planting and investigating this cultivar were obtained from Hebei Agricultural University and the National Medium-term Gene Bank of Cotton in China, and the collection and research of this cultivar have complied with the Convention on the Trade in Endangered Species of Wild Fauna and Flora.

### Fiber analysis

Seven replicates were taken for each treatment (NI and WD). For each replication, mature seed fibers were randomly sampled from 20 naturally-open bolls on the middle section of cotton plants. After drying, seed cotton was treated with a roller gin (MPSY-20, River Machinery Factory, Xinxiang, Henan, China) for separation of lint and seed, which were weighed to determine lint percentage (LP). Weight of 100 seeds was expressed as seed index (SI). The lint index (LI) was calculated by the formula: LI = (SI × LP)/(1-LP). The fiber qualities were determined with an HVI-MF 100 instrument (User Technologies, Inc., USTER, Switzerland) at the Supervision, Inspection and Testing Center of Cotton Quality, Ministry of Agriculture, Anyang, China. The data were analyzed (*P*-values < 0.05 was considered statistically significant) by unpaired *t*-test using GraphPad Prism 8.0.2 software (GraphPad Software, San Diego, USA).

### Fiber sample collection, RNA isolation, library construction and sequencing

Flowers were tagged on the day of flowering as 0 DPA. Ovules (0 and 5 DPA) and fibers (10, 15, 20, 25, 30 and 35 DPA) were collected, frozen immediately in liquid nitrogen and stored at − 80 °C. Samples (ovules and fibers) from five independent plants within each treatment group served as one biological replication. Total RNA was isolated using the EASYspin Plant RNA Kit (Aidlab, Beijing, China). Libraries were constructed using the NEBNext® Ultra™ Directional RNA Library Prep Kit for Illumina® (NEB, USA) according to the manufacturer’s instructions. Strand-specific sequencing was performed on the Illumina HiSeq 4000 platform (paired-end 150-bp reads).

### mRNA and lncRNA identification and differential expression analysis

All raw data were processed by removing reads containing adapter or ploy-N and reads with low quality. The clean reads were aligned to the *G. hirsutum* TM-1 genome (NAU-NBI v1.1) [43] using TopHat v2.0.9 [44]. The mapped reads for each sample were assembled by Cufflinks v2.1.1 in a reference-based approach to identify mRNA transcripts (fragments per kilobase per million mapped reads, FPKM ≥1) [45]. Then, five steps were adopted to screen out lncRNAs from assembled transcripts: (1) transcripts with one exon, low expression, and low credibility were removed; (2) transcripts with length < 200 bp were eliminated; (3) transcripts that overlapped with annotated exons in the database were filtered out; (4) transcripts with FPKM < 0.5 were removed (however for transcripts with single exon, the threshold value was 2); (5) finally, transcripts with protein coding potential by the Coding Potential Calculator with NCBI eukaryotic protein database (*E*-value <1e-10) and Pfam Scan (v1.3) (default parameters) were excluded [46, 47]. FPKMs of both mRNAs and lncRNAs in each sample were calculated using Cuffdiff (v2.1.1), which also provides statistical routines for determining differential expression using a model based on the negative binomial distribution [45]. Corrected *P*-value < 0.05 and the absolute value of log2(FPKM_WD_/FPKM_NI_) < 1 were set as the threshold for significantly differential expression when processing the data with Cuffdiff.

### Real-time quantitative PCR (qPCR) analysis

Total RNA was reverse transcribed into cDNA using TransScript® One-Step gDNA Remover and cDNA Synthesis SuperMix (TransGen Biotech, China), according to the manufacturer’s instructions. qPCR was conducted on a QuantStudio™ 1 Real-Time PCR system (Thermo Fisher Scientific, USA) with TransStart Top Green qPCR SuperMix (+Dye I/+Dye II) (TransGen Biotech, China). The expression levels of mRNAs and lncRNAs were normalized by *GhUBQ7* (ubiquitin, Genbank accession No. DQ116441.1) using the 2^*-∆∆CT*^ method [48]. The primers used in the study are listed in Table S1.

### Functional analysis of differentially expressed genes (DEGs) and lncRNAs (DERs)

The functions of lncRNAs were predicted according to the functional annotations of their potential target genes, which may be regulated by lncRNAs using two patterns: *cis-*acting (genomic colocation) and *trans*-acting (coexpression). The coding genes in the 100 kb up- or down-stream of lncRNAs were identified as colocalized genes. Coexpressed genes were predicted by the correlation in the expression between lncRNAs and coding genes (Pearson’s correlation coefficient ≥ 0.95 or ≤ − 0.95). KOBAS 3.0 was used to test the statistical enrichment of genes in GO and KEGG pathways. A corrected *P*-value ≤0.05 was considered significantly enriched.

### Interaction prediction for lncRNA-miRNA-mRNA

The sequences of premiRNAs (precursor stem-loop molecules) and mature miRNAs in *G. hirsutum* were retrieved from the PNRD websites (http://structuralbiology.cau.edu.cn/PNRD/index.php) [49]. The targets (mRNAs and lncRNAs) of miRNAs were predicted using the psRNATarget online analysis tool (http://plantgrn.noble.org/psRNATarget/analysis) [50]. The potential interaction for lncRNA-miRNA-mRNA was constructed by (i) analyzing the same target miRNA for both lncRNA and mRNA and by (ii) evaluating the correlation in the expression between lncRNA and mRNA (Pearson’s correlation coefficient ≥ 0.95 or ≤ − 0.95).

## Results

### Water deficit reduces cotton fiber qualities

**Fig. 1:**
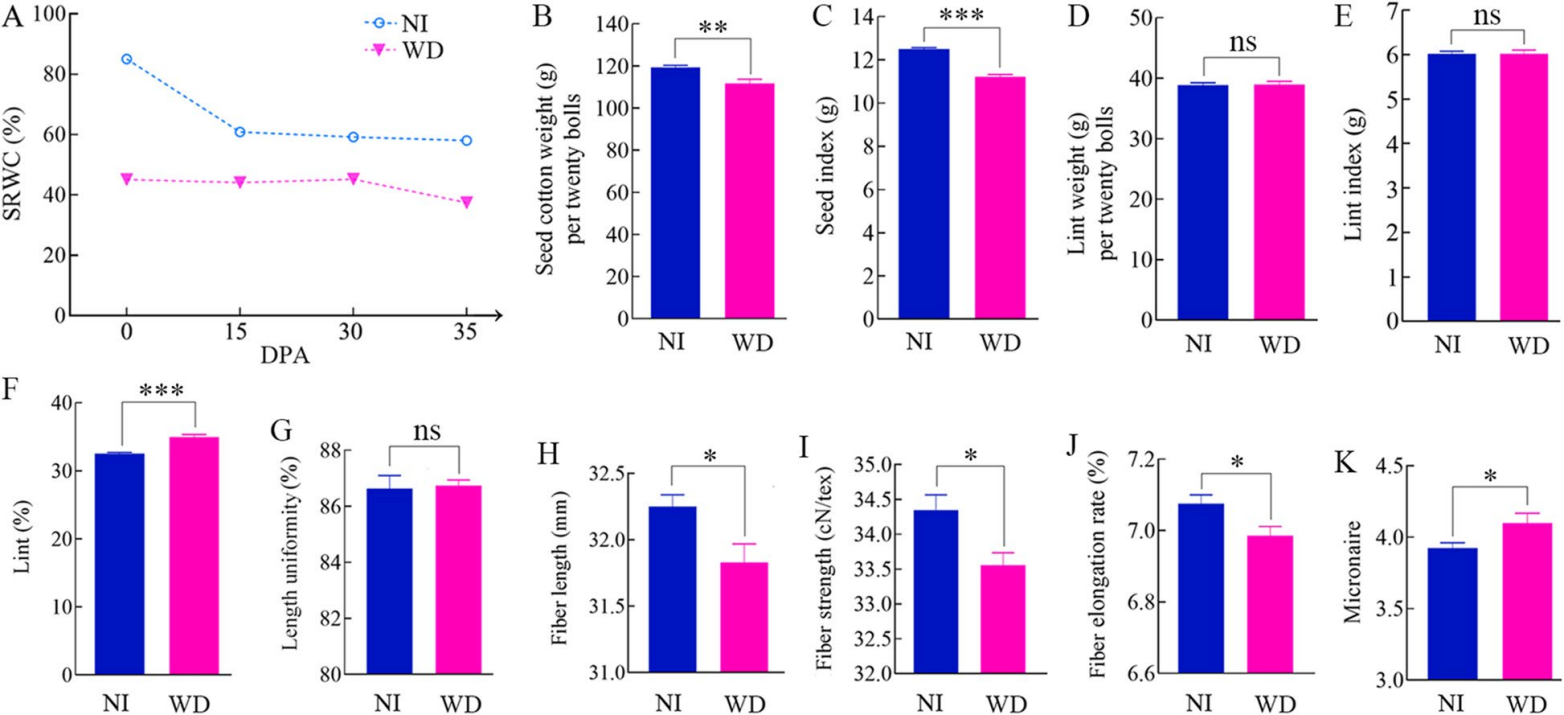
Water deficit caused a significant reduction in fiber quality. **A** SRWC in the NI-field and WD-field at 0, 15, 30 and 35 DPA. Seed cotton weight (**B**), seed index (**C**), lint weight (**D**), lint index (**E**), lint (**F**), length uniformity (**G**), fiber length (**H**), fiber strength (**I**), fiber elongation rate (**J**), and micronaire (**K**), for NI-treatments were compared with WD-treatments. Data represent the mean ± SE of seven biological replicates. Fibers for each replication were sampled from 20 naturally-open bolls on the middle section of cotton plants. Confidence levels were tested by unpaired *t*-test (*, *P* < 0.05; ns, not significant)

Two cotton fields received different treatments, NI and WD, at the beginning of the flowering stage. The SRWC in the NI-field (85.06%) was nearly twice as high as that in the WD-field (45.09%) at 0 DPA. During the following days, the SRWC in WD (37.47 ~ 45.17%) remained lower than that in NI (58.02 ~ 60.91%) (Fig. 1A) until cotton boll maturation began at 35 DPA. After another 30 days, fully mature cotton fibers were collected for weighing and quality determination. The seed cotton weight of the WD-group was significantly lower than that of the NI-group (Fig. 1B). The SI of the WD-group was also significantly lower than that of the NI-group (Fig. 1C), but there was no significant difference in lint weight (Fig. 1D) or LI (Fig. 1E) between the two groups. These results indicated that water deficit affected cotton seed weight but did not significantly affect fiber weight. Thus, water deficit-induced seed weight reduction resulted in a significant increase in LP (Fig. 1F). The uniformity ratio of fiber was not significantly reduced in WD compared with NI (Fig. 1G). However, water deficit caused a significant reduction in fiber length (Fig. 1H), strength (Fig. 1I) and elongation rate (Fig. 1J), and a dramatic increase in micronaire value (Fig. 1K). These results indicated that water deficit during cotton fiber development could lead to lower fiber qualities.

### Overview of fiber transcriptomes

**Fig. 2:**
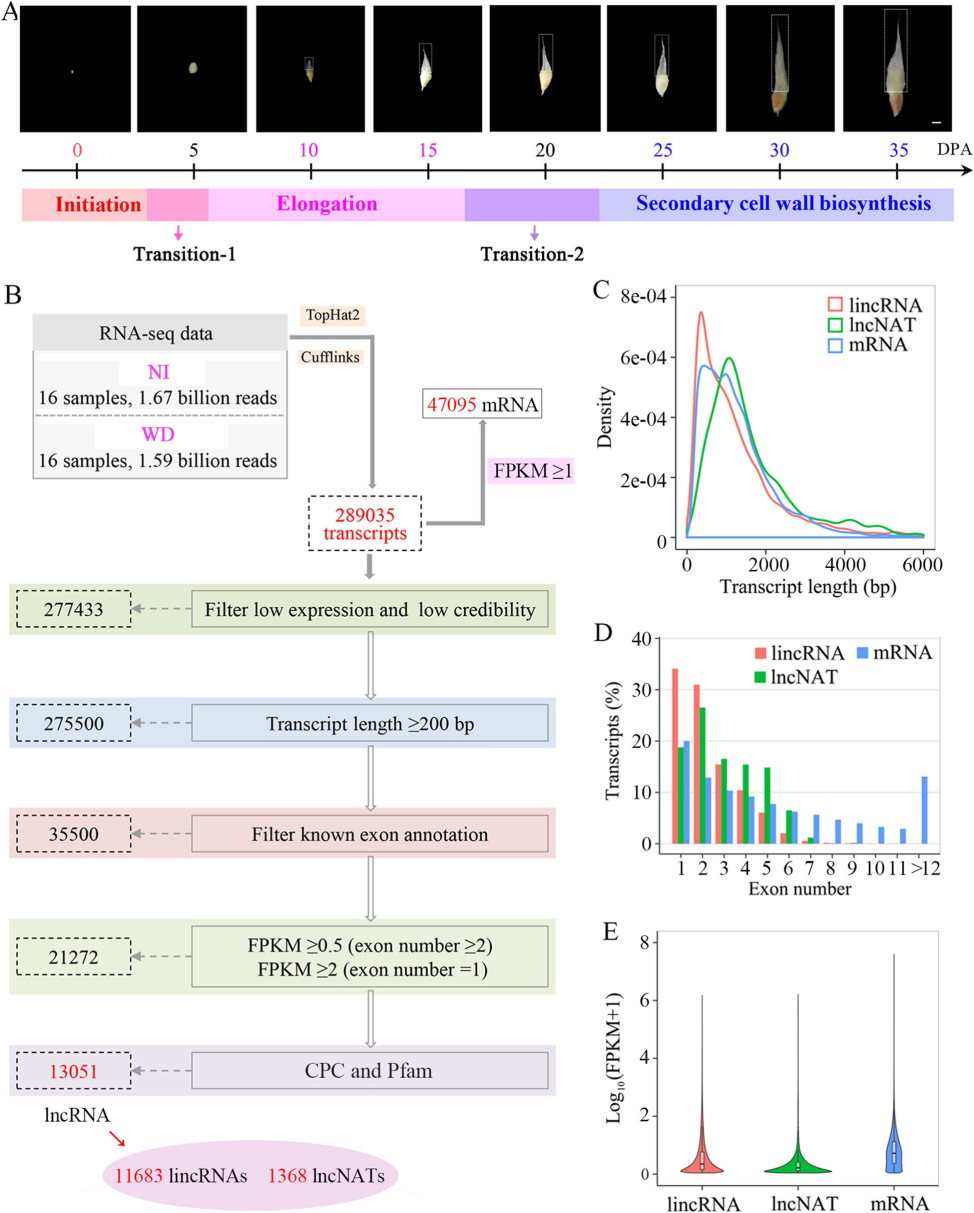
Identification and characterization of mRNAs and lncRNAs in *G. hirsutum* fibers. **A** Representative images of individual seeds with attached fibers from 0 DPA to 35 DPA. Cotton fibers undergo three major sequential and overlapping developmental stages before maturity, including initiation, elongation and secondary cell wall biosynthesis. Transition-1 and -2 are two fiber developmental transition stages, which are from initiation to elongation and from elongation to secondary cell wall biosynthesis, respectively. The scale bars in all panels are 0.5 cm. **B** The pipeline of mRNAs and lncRNAs identification. **C** Length density distributions of lincRNAs, lncNATs and mRNAs. **D** Exon number per transcript of lincRNAs, lncNATs and mRNAs. **E** FPKM distributions of lincRNAs, lncNATs and mRNAs

To explore the transcriptional regulation of cotton fiber development under water deficit, the fiber transcriptomes of *G. hirsutum* from FI to SCWB were sequenced using RNA-seq. cDNA libraries from eight time points were constructed, which included 0, 5, 10, 15, 20, 25, 30 and 35 DPA. The flowering day was designed as 0 DPA, which represents FI. Both 10 and 15 DPA represent FE. To evaluate the SCWB, three time points including 25, 30 and 35 DPA were chosen. In addition, 5 and 20 DPA represent fiber developmental transitions (FDT1 and FDT2), which are stages from FI to FE and from FE to SCWB, respectively (Fig. 2A). Approximately 3.22 billion clean reads were screened out from 3.26 billion raw reads, varying from 83 to 116 million reads per library. The mapping rates of each library to the reference genome of *G. hirsutum* TM-1 ranged from 80.72% to 89.27% (Table S2). The correlation coefficients for two biological replicates at each time point were all above 0.85 (Table S3), indicating that the RNA-seq data have high reproducibility.

**Fig. 3:**
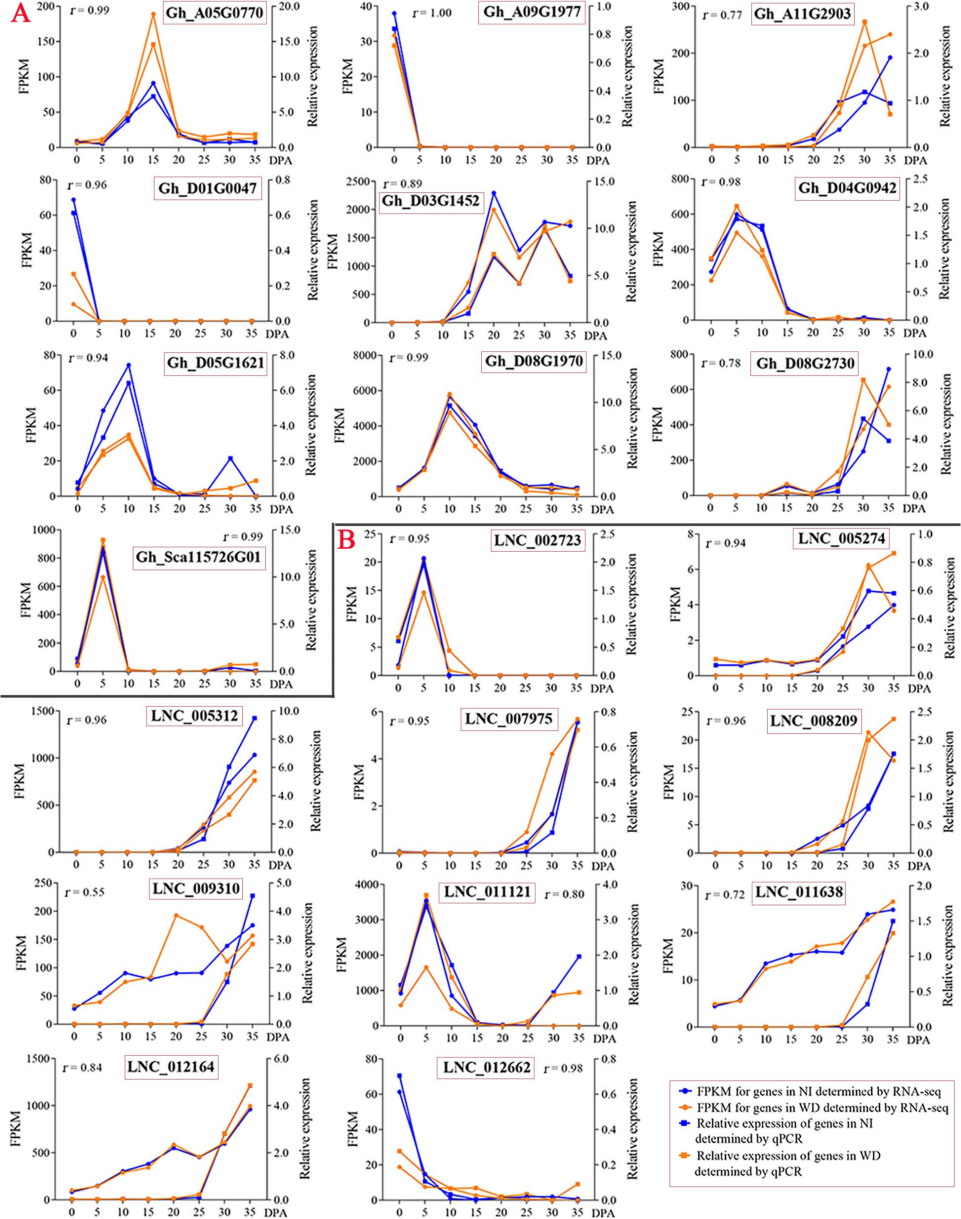
Confirmation of the expression patterns of mRNAs (**A**) and lncRNAs (**B**) using qPCR. Ten mRNAs and ten lncRNAs were randomly selected for expression analysis during the fiber developmental stages of *G. hirsutum* ND13 treated with NI and WD. The correlation of relative expression for mRNAs and lncRNAs measured by RNA-seq and qPCR was estimated with *r*-values. *UBQ7* was used as the reference gene. Gene (mRNA) IDs are shown in the genome of *G. hirsutum* TM-1 (NAU-NBI v1.1), including Gh_A05G0770 (17.3 kDa class I heat shock protein), Gh_A09G1977 (1-aminocyclopropane-1-carboxylate oxidase homolog 1), Gh_A11G2903 (ABC transporter G family member 2), Gh_D01G0047 (Protein RADIALIS-like 6), Gh_D03G1452 (Tubulin beta-7 chain), Gh_D04G0942 (No annotation), Gh_D05G1621 (No annotation), Gh_D08G1970 (Probable aquaporin PIP1-2), Gh_D08G2730 (Bidirectional sugar transporter SWEET15), and Gh_Sca115726G01 (Aspartic proteinase nepenthesin 1)

In total, 47,095 mRNAs (putative protein-coding genes) were identified with FPKM ≥1 and annotated according to the reference genome of *G. hirsutum* (Table S4). After multistep filtering, 13,051 high-confidence lncRNAs were identified, including 11,683 lincRNAs (89.52%) and 1368 lncNATs (10.48%) (Fig. 2B, Table S5). The transcript length of lncNATs (mean = 1883 nt) was significantly longer than that of lincRNAs (mean = 1327 nt) and mRNAs (mean = 1307 nt) (Fig. 2C). Most lincRNAs and lncNATs contained fewer than 6 exons, while mRNAs contained various numbers of exons (Fig. 2D). The overall expression levels of both lincRNAs and lncNATs were lower than those of mRNAs (Fig. 2E). To validate the reliability of the transcriptome, 10 mRNAs and 10 lncRNAs were randomly selected for expression analysis by qPCR. For most mRNAs and lncRNAs, the linear regression analysis revealed a positive correlation between the transcriptome data and the results from qPCR with *r*-values (Fig. 3), suggesting the high quality of transcriptomes. Only one lncRNA, LNC009310, showed a relatively low correlation between transcriptome and qPCR analysis (*r* = 0.55), likely due to the low expression of lncRNAs [51].

### Identification of differentially expressed genes (DEGs) in cotton fibers between NI and WD

**Fig. 4:**
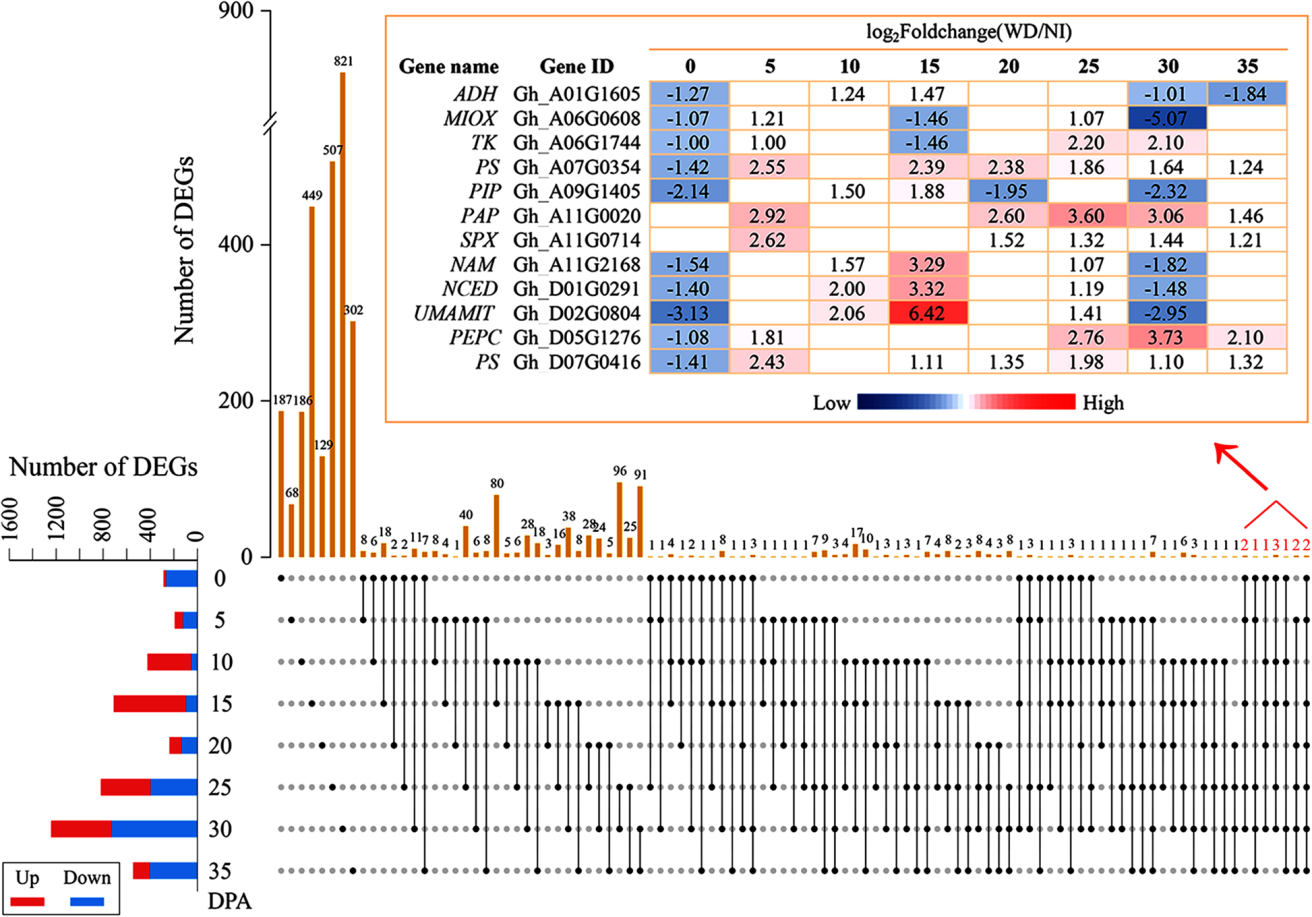
Identification and characterization of DEGs between NI-treated and WD-treated cotton fibers. Twelve genes were differentially expressed at ≥5 time points, including *ADH* (alcohol dehydrogenase), *MIOX* (*myo*-inositol oxygenase), *TK* (thymidine kinase), *PS* (phosphate starvation-induced gene), *PIP* (plasmamembrane intrinsic protein), *PAP* (purple acid phosphatase), *SPX* (SYG1-Pho81-XPR1 domain-containing protein), *NAM* (no apical meristem), *NCED* (9-cis-epoxycarotenoid dioxygenase), *UMAMIT* (usually multiple acids move in and out transporter) and *PEPC* (phosphoethanolamine/phosphocholine phosphatase)

A total of 3427 DEGs with at least a twofold expression change (FPKM_WD_/FPKM_NI_, corrected *P*-value<0.05) were identified (Fig. 4, Table S6). During the stages of FI, SCWB, and FDTs, most DEGs were downregulated, whereas approximately 87% of DEGs were upregulated at the FE stage. At the two FDT stages, fewer DEGs were identified compared with the other three stages of fiber development. The maximum number of DEGs was identified at the SCWB stage with 2265, among which the greatest number of genes were differentially expressed at 30 DPA. Furthermore, the expression specificity of these DEGs at different stages of cotton fiber development was observed. Many genes were only differentially expressed at one time point, suggesting that they have time-specific expression in fibers under the stress of water deficit. For example, 821 DEGs were shown to be differentially expressed only at 30 DPA. No DEGs appeared at all 8 time points, but 12 genes were differentially expressed at ≥5 time points, including *ADH* (alcohol dehydrogenase), *MIOX* (*myo*-inositol oxygenase), *TK* (thymidine kinase), *PS* (phosphate starvation-induced gene), *PIP* (plasmamembrane intrinsic protein), *PAP* (purple acid phosphatase), *SPX* (SYG1-Pho81-XPR1 domain-containing protein), *NAM* (no apical meristem), *NCED* (9-cis-epoxycarotenoid dioxygenase), *UMAMIT* (usually multiple acids move in and out transporter) and *PEPC* (phosphoethanolamine/phosphocholine phosphatase), indicating that they maintain an intense response to the stress of water deficit.

### Functional classification of DEGs

**Fig. 5:**
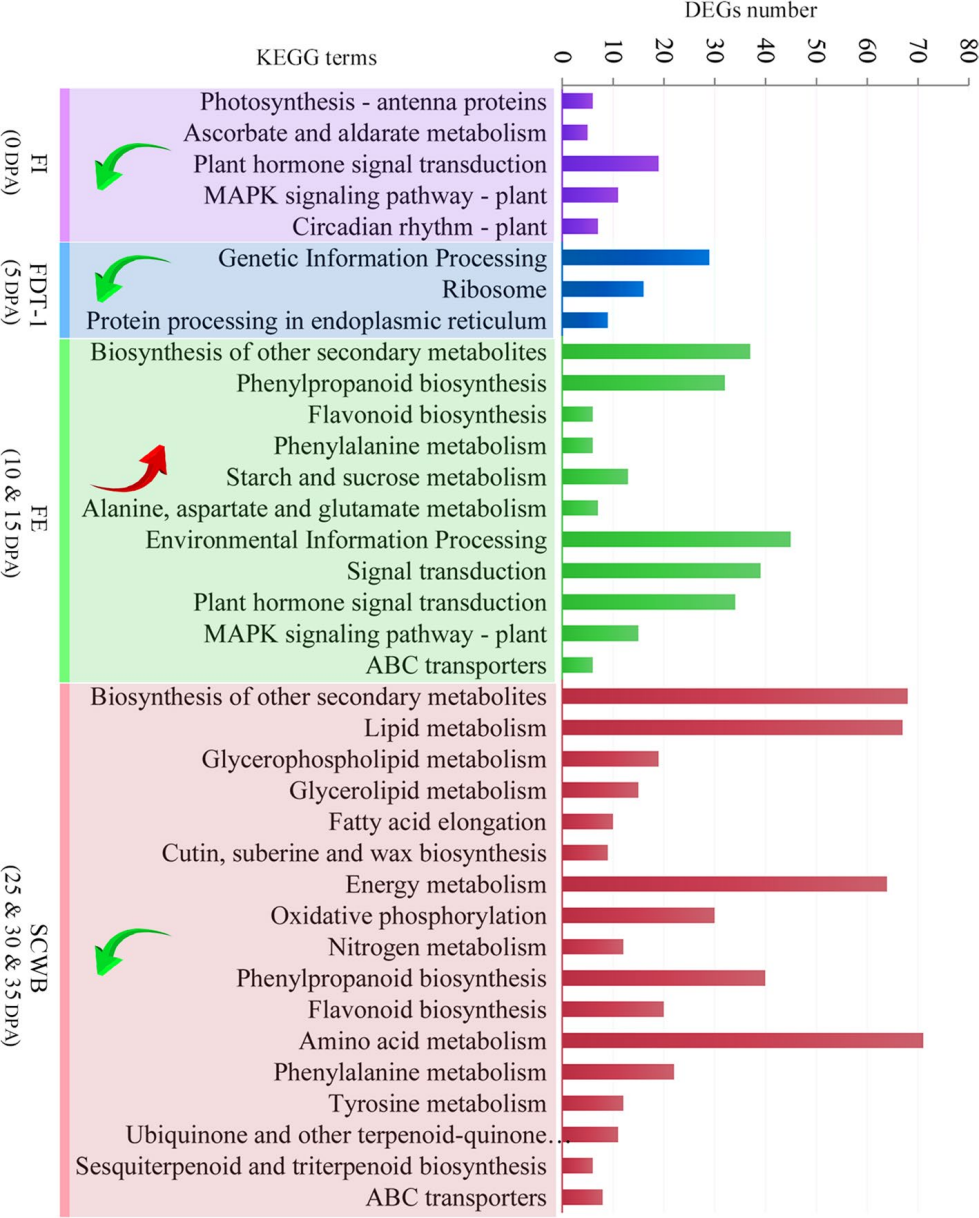
The significantly enriched KEGG pathways of DEGs between NI-treated and WD-treated cotton fibers. The overall trends of upregulation and downregulation for DEGs are indicated by red and green arrows, respectively

To further characterize the functional consequences of gene expression changes in cotton fiber cells associated with water deficit, pathway enrichment analyses for DEGs were performed using the KEGG database (Fig. 5). At the FI stage, DEGs were significantly enriched in 5 pathways, especially “plant hormone signal transduction” and “photosynthesis-antenna proteins”. At the FE stage, DEGs were significantly enriched in 11 pathways, mainly “plant hormone signal transduction” and “phenylpropanoid biosynthesis”. At the SCWB stage, “plant hormone signal transduction” was no longer enriched, and 17 pathways related to metabolism and 1 pathway related to ABC transporters were significantly enriched. The pathways of “lipid metabolism”, “energy metabolism”, “amino acid metabolism” and “phenylpropanoid biosynthesis” are possibly involved in the further development of fiber and the cell wall. Only three pathways related to genetic information processing were enriched at FDT1, and no pathway was enriched at FDT2. In addition, almost all DEGs involved in enriched pathways for FI, FDT1 and SCWB were significantly downregulated, suggesting that these biological processes were suppressed under the stress of water deficit. Instead, almost all DEGs involved in pathways at the FE stage were significantly upregulated, suggesting that these biological processes were activated.

### Different expression profiles of lncRNAs in cotton fibers under water deficit

**Fig. 6:**
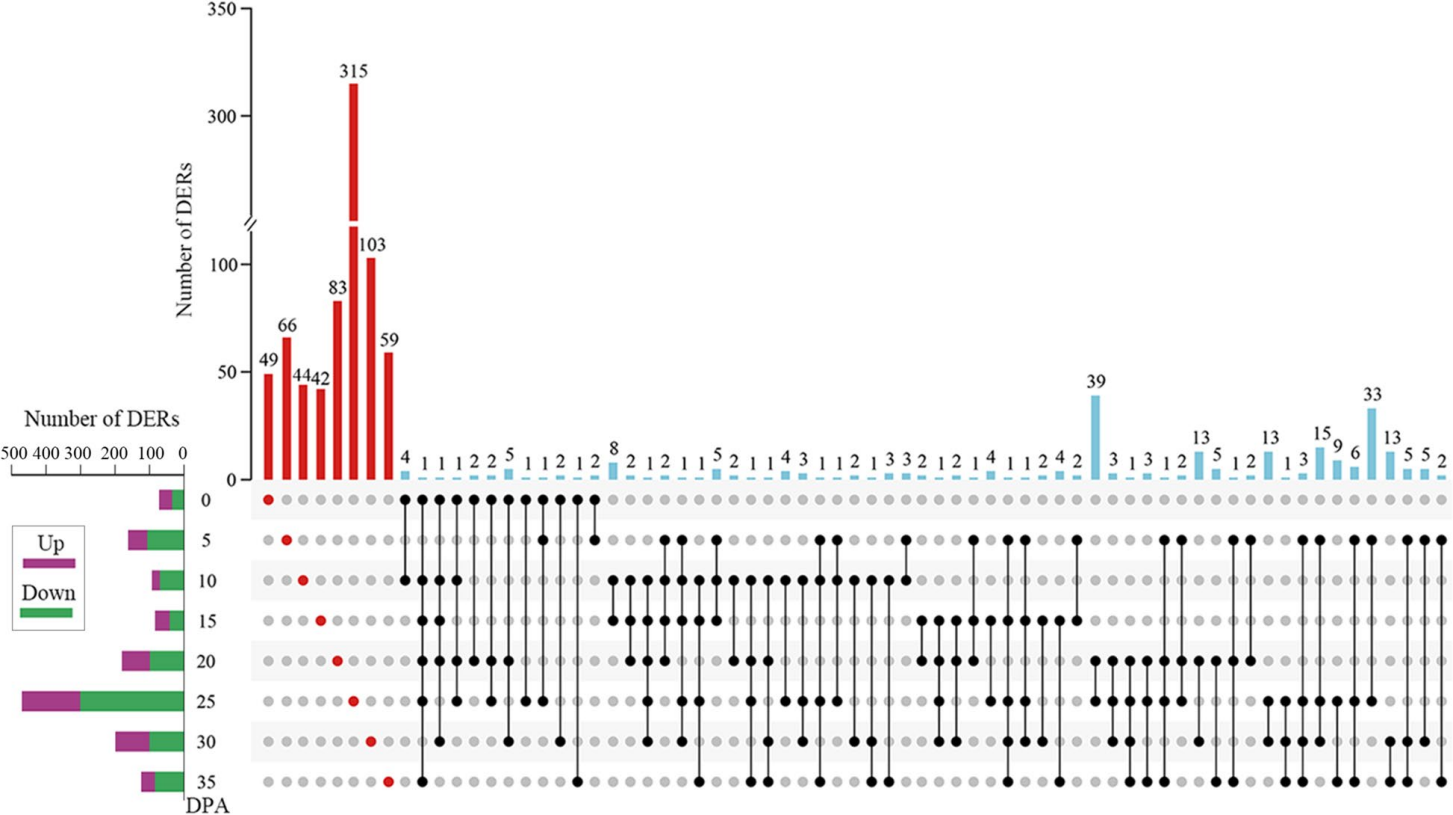
Identification and characterization of DERs between NI-treated and WD-treated cotton fibers. Green bars for downregulated DERs. Purple bars for upregulated DERs. Red bars and dots for DERs that were only shown at one time point

Under water deficit stress, a total of 1021 lncRNAs were differentially expressed (DERs) (Table S7), of which the majority were significantly downregulated (Fig. 6). In addition, the majority of DERs were only expressed at a specific time point (Fig. 6), showing similar expression characteristics to DEGs. Up to 700 DERs were identified at the SCWB stage, and many lncRNAs were specifically and differentially expressed at 25 DPA, as many as 315 in total, suggesting that lncRNAs mainly play a role in SCWB for fibers in response to water deficit.

### DEGs expression were potentially regulated by DERs

**Fig. 7:**
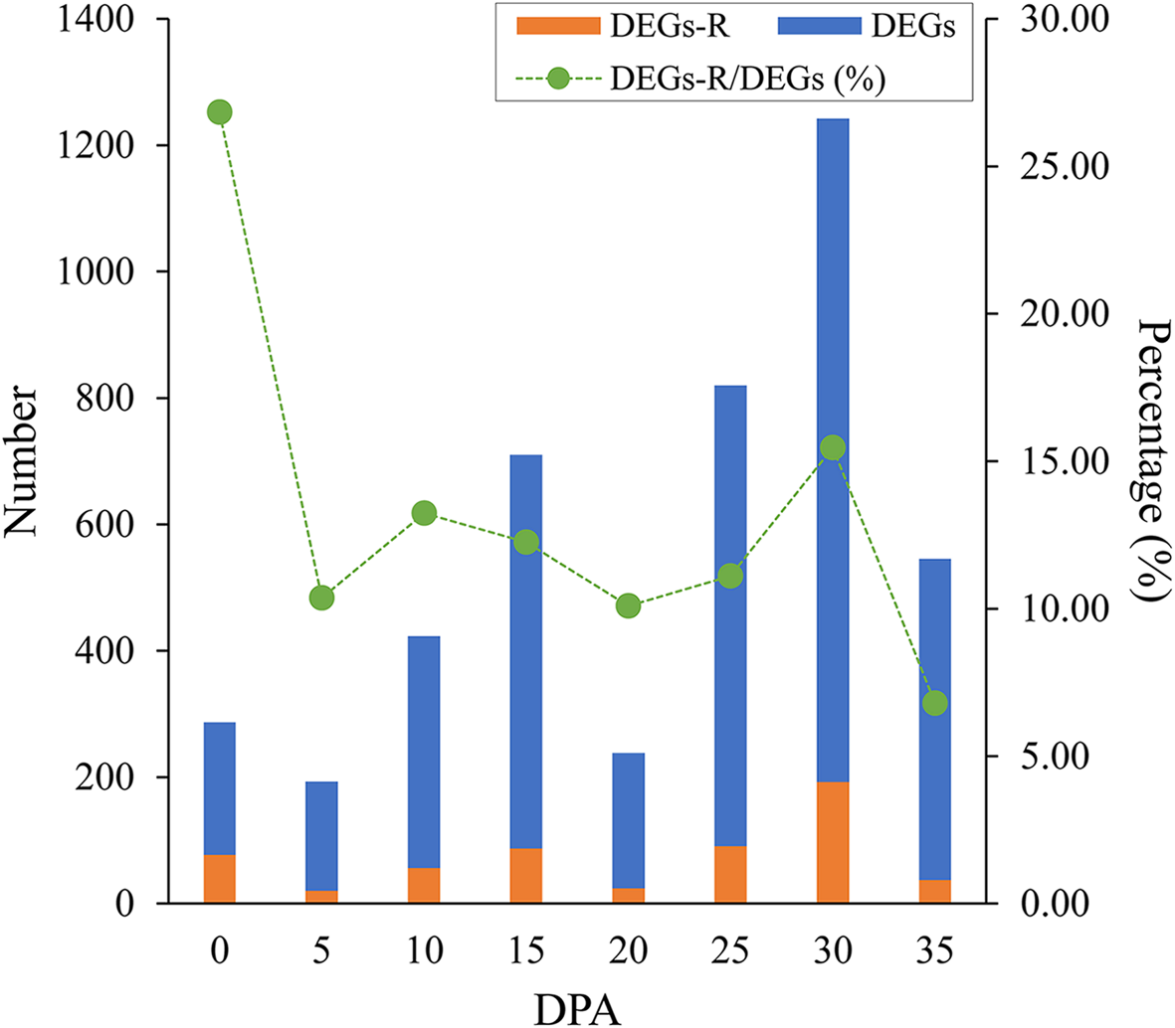
DEGs-R predicted by gene coexpression and genomic colocation analysis for DEGs and DERs. Blue bars for DEGs. Orange bars for DEGs-R. Green dots for the percentages of DEGs-R/DEGs

**Fig. 8:**
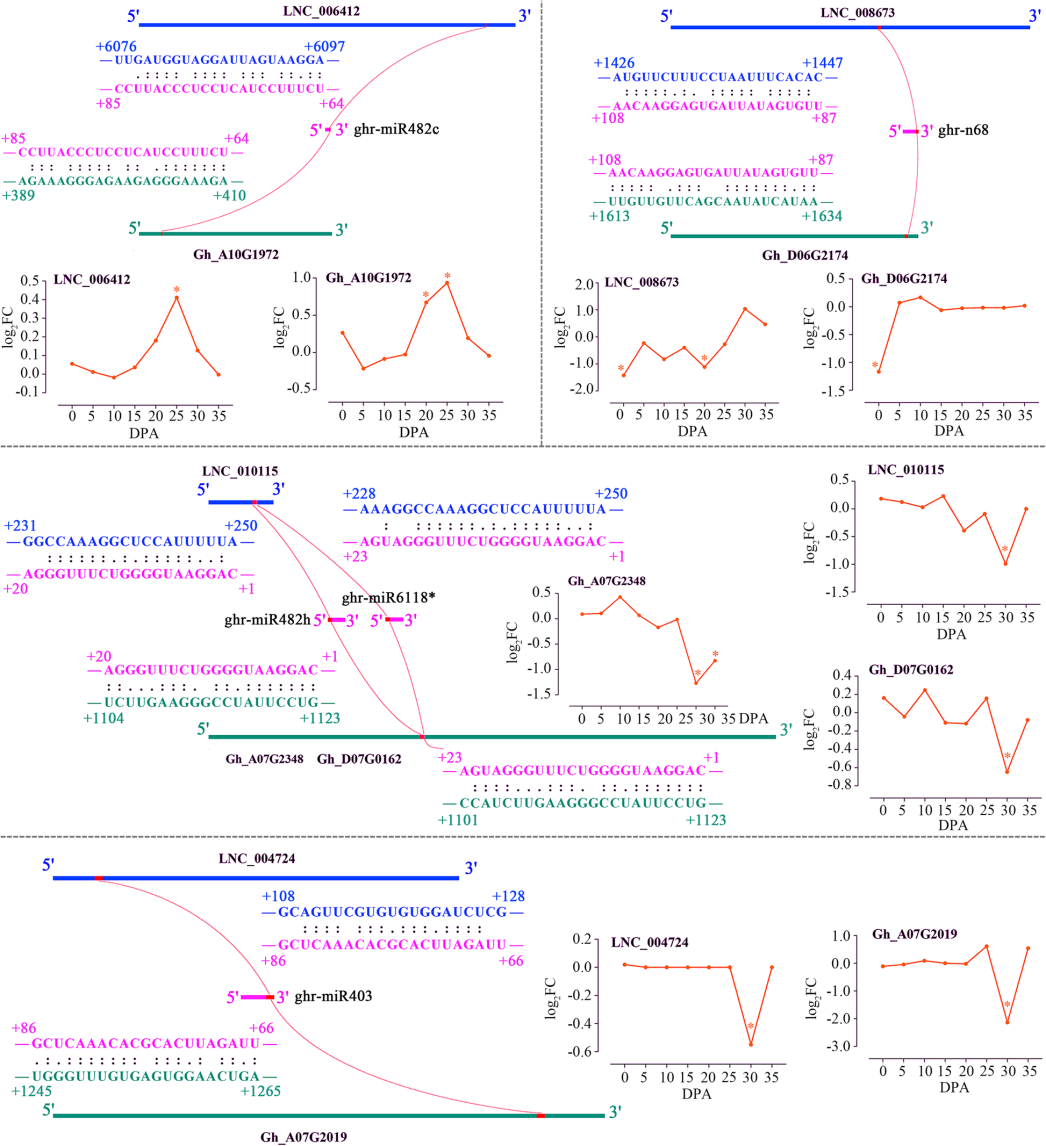
Prediction of the interactions between lncRNAs, miRNAs and mRNAs by forming RNA-RNA duplexes. miRNA-directed target mRNA degradation was potentially regulated by forming a lncRNA-miRNA duplex. Gene (mRNA) IDs are shown in the genome of *G. hirsutum* TM-1 (NAU-NBI v1.1), including Gh_A10G1972 (DEAD-box ATP-dependent RNA helicase 42), Gh_D06G2174 (protein of unknown function), Gh_A07G2348 and Gh_D07G0162 (LRR receptor-like serine/threonine-protein kinase), and Gh_A07G2019 (UDP-glycosyltransferase 88F3). The expression of lncRNAs and mRNAs is shown with log_2_FoldChange (WD/NI)

By analysis of coexpression and genomic colocation a total of 540 DEGs were predicted to be potentially regulated by DERs (Table S8), accounting for approximately 15.76% of all DEGs. As shown in Fig. 7, the largest number of DEGs regulated by DERs (DEGs-R) was found at the SCWB stage (25, 30 and 35 DPA). However, according to the proportion calculation, the FI stage (0 DPA) had a larger proportion of DEGs-R (25.44%). DEGs-R were used for further GO enrichment analysis (Table S9). GO terms “cell wall organization or biogenesis” and “cell wall macromolecule metabolic process” in the biological process category were enriched, suggesting that lncRNAs targeting mRNA mainly regulate cell wall development. GO terms of “DNA packaging complex”, “protein-DNA complex”, “nucleosome”, “chromatin” and “chromosomal part” in the cellular component category were enriched, indicating that lncRNAs play regulatory roles in the nucleus. Only one GO term in the molecular function category was enriched (“Hydrolase activity”), showing that lncRNAs mainly function by regulating hydrolases. In addition, four pairs of regulatory relationships between DEGs and DERs mediated by miRNAs were predicted (Fig. 8), including LNC_006412::ghr-miR482c::Gh_A10G1972, LNC_008673::ghr-n68::Gh_D06G2174, LNC_010115::ghr-miR482h/ghr-miR6118*::Gh_A07G2348/Gh_D07G0162, and LNC_004724::ghr-miR403::Gh_A07G2019. These DERs may potentially combine with miRNAs, which also possibly interact with DEGs, using sequence complementation containing several mismatches.

## Discussion

Water deficiency is one of the most impactful stresses worldwide and has long been prevalent in many countries, leading to reductions in cotton productivity and fiber quality. This negative effect varies depending on cotton growth stages and the intensity of water deficit [52]. SIF is always applied as an effective measure for guaranteeing and increasing cotton yield in the YRB regions of China. Here, our results strongly support the importance of SIF in cotton fiber development. From 0 DPA (bloom) to 35 DPA (fiber maturity), the SRWC of cotton fields that received SIF was significantly higher than that of cotton fields without SIF (Fig. 1A). Therefore, the cotton plants grown in the field without SIF were considered to be subjected to water deficit stress throughout cotton fiber development. As expected, water deficit caused a significant reduction in seed cotton yield (Fig. 1B and C). However, lint yield did not significantly decrease because of the water deficit (Fig. 1D and E). As a result, there was a significant increase in the percentage of lint (Fig. 1F). Therefore, it can be concluded that the loss of seed cotton yield is mainly due to the reduction in seed weight, which is caused by non-SIF. Furthermore, the quality of fibers from WD was compared with that from NI. Water deficit caused a significant reduction in fiber length (Fig. 1H), fiber strength (Fig. 1I), elongation rate (Fig. 1J) but a dramatic increase in micronaire (Fig. 1K), suggesting that fiber development is very sensitive to water deficit. In addition, micronaire is an indicator of air permeability and universally used for assessing fiber maturity (degree of secondary cell-wall development) and fineness [53]. Here, fully mature cotton fibers (65 DPA) were collected for weighing and quality determination. Thus, the micronaire value mainly represents the thickness of the fiber. Additionally, water deficit usually induces the thickening of cell walls, which is an important adaptation to increase plant tolerance to water loss [54]. Therefore, it can be inferred that water deficit makes cotton fibers thicker. Fibers with micronaire values that are too high or too low are undesirable from the point of view of spinning and yarn evenness. Micronaires have been shown to increase or decrease with irrigation changes [55, 56]. Here, water deficit significantly increased the micronaire value of ND13. Therefore, the expected micronaires might be obtained by properly controlling SIF in the future to meet the demand of the cotton industry.

To further our knowledge of the molecular mechanisms underlying fiber cells response to water deficit, a genome-wide identification and characterization of water deficiency-responsive genes and lncRNAs was carried out in this study. At the FI and FE stages, the pathway “plant hormone signal transduction” was enriched (Fig. 5), suggesting that water deficit affected the regulatory networks controlled by various hormones that are necessary for fiber initiation and elongation. According to the number of DEGs, auxin, ethylene and ABA are the most important signal regulatory molecules for cotton fibers in response to water deficit. This is consistent with previous reports that these hormones are involved not only in fiber development but also in plant drought resistance [57–59]. At the FE stage, up to 32 DEGs were involved in “phenylpropanoid biosynthesis”, which participates in the biosynthesis of many plant cell wall phenolic products, such as lignins, flavonoids, suberins, and cutins [60, 61]. The significantly upregulated expression of these DEGs may affect fiber cell expansion and prematurely end cell elongation, ultimately leading to a significant reduction in fiber length. However, at the SCWB stage, DEGs involved in “phenylpropanoid biosynthesis” were changed to be significantly downregulated, which may reduce cell wall thickening and lignin deposition [62]. Although the content of lignin and lignin-like phenolics is minor in cotton fibers, recent data suggest that these ingredients are strongly associated with fiber strength and elongation [62–64].

In addition, the expression of most DEGs showed temporal specificity; that is, they were expressed only at one point in time. However, 12 genes were differentially expressed at more than 5 time points (Fig. 4), suggesting that they can intensely and persistently respond to water deficit. MIOX is known to balance the concentrations of *myo*-inositol and UDP-GlcA (UDP-glucuronic acid). *Myo*-inositol plays an important role in drought tolerance by scavenging reactive oxygen species, decreasing the loss of chlorophyll for photosynthesis, and improving antioxidant enzyme activity [65, 66]. UDP-GlcA is the precursor for UDP-xylose, which is a critical component of cell wall polysaccharides, such as pectin and hemicellulose [67]. PIPs are major facilitators that conduct water and/or other molecules across cell membranes. Thus, PIPs are usually responsive to drought stress and play pivotal roles in plant drought resistance by regulating the transcellular transport of water [68]. Meanwhile, PIPs can selectively form primary aquaporin isoforms to meet the requirements for rapid elongation of fibers [69]. Thus, *MIOX* and *PIP* in cotton fibers were differentially and persistently expressed, suggesting that they are difunctional genes involved in both fiber development and drought resistance, and can be potentially used in breeding to improve cotton resistance to water deficit.

Previously, thousands of lncRNAs have been identified and proposed to have functions in fiber development [23, 25], resistance to *V. dahliae* [29, 30], response to drought [27] and salt [28]. Here, our study provides the first comprehensive identification of lncRNAs in fiber cells of *G. hirsutum* under water deficit conditions. Up to 700 DERs (approximately 68.56% of the total DERs) were identified at the SCWB stage (Fig. 6), which is key for determining fiber length and strength. In addition, more than 300 DERs were specifically expressed at 25 DPA. These results suggest that the expression of lncRNAs in fibers changes in response to water deficit and differs significantly depending on fiber development. Understanding on the mechanisms of lncRNA action in plants remains limited and a major challenge [32]. LncRNAs have no discernable protein coding potential, or can encode only small peptides, but often result in functional RNAs involved in a wide range of molecular processes including but not limited to all steps of gene expression involving nucleic acids from chromatin modifications to translation [70]. Cotton lincRNA DAN1, a well-known example for transcriptional regulation, can bind DNA sequences containing AAAG motifs hence silencing of DAN1 increased cotton drought tolerance by regulating auxin responsive genes with AAAG motifs [71]. Target mimicry is a generally accepted and prediction available post-transcriptional regulating mechanism by which lncRNA affect mRNA expression by regulating miRNA activity [35]. LncRNAs are called miRNA “sponges” that sequester miRNAs with imperfect base complementarity. In cotton, lncRNA354 is negatively related to salt tolerance by regulating ARF genes through miR160b [72]. Here, with WD, 540 DEGs were predicted to be potentially regulated by DERs by analysis of coexpression and genomic colocalization with mRNA. Furthermore, four pairs of regulatory relationships between DEGs and DERs mediated by miRNAs were predicted (Fig. 8). These four lncRNAs potentially interact with a miRNA by forming an lncRNA-miRNA duplex, which functions as RNA interference (RNAi), and as a result, miRNA-targeted mRNA is normally translated into a functional protein [32]. MiR403 has been reported to be involved in plant drought [73–75], heat, salt and cadmium stress response in a tissue associated manner [73]. Additionally, miR403 has an antiviral role by controlling the expression of AGO2 (Argonaute 2) [76]. In our study, the putative target gene of ghr-miR403, *UGT88F3* (UDP-glycosyltransferase 88F3, Gh_A07G2019) is likely involved in fiber development and osmotic stress [77]. MiR482 is an ancient microRNA family present in all land plants. In tomato, overexpression of miR482c induced enhanced susceptibility to late blight while knock out miR482c and miR482b simultaneously enhanced resistance to late blight and the effect was stronger than silencing miR482b alone, possibly by regulating expression levels of genes encoding for proteins with nucleotide binding sites and leucine-rich repeat (NBS-LRR) domains and ROS levels [78, 79]. As in tomato, cotton plants are able to induce expression of NBS-LRR defence genes by suppressing the miR482-mediated gene silencing pathway upon fungal pathogen attack [80]. Particularly, miR482c might participate in cotton growth and abiotic stresses including drought stress response as a potential regulator [81]. As sequence variation induced target and mechanism variation, the function of members in miR482 family differ possibly from each other [78, 82]. *RH42* (DEAD-box ATP-dependent RNA helicase 42, Gh_A10G1972) plays an essential role in DNA and RNA metabolism such as transcription, replication and repair [83]; *ERECTA* (LRR receptor-like serine/threonine-protein kinase, Gh_A07G2348 and Gh_D07G0162) has been shown to regulate plant flowering and transpiration efficiency in part via effects on epidermal cell expansion, cell-cell communication, and stomatal density [84, 85]. Gh_D06G2174 with a DUF616 domain (protein of unknown function) has not been described and annotated in the genomic database of *G. hirsutum*. Although DEGs potentially regulated by lncRNAs and miRNAs have been successfully found here, more experiments are needed to further confirm the interactions between them.

## Conclusions

Water deficit during cotton fiber development caused a significant reduction in cotton seed yield and fiber quality. Through the identification and functional classification of DEGs and DERs in cotton fibers between NI and WD, a valuable platform for revealing the molecular mechanism of cotton against water deficit was provided. In addition, potential functions for some lncRNAs regulating mRNA transcription were predicted, which provides valuable information to further characterize their functions.

## Supplementary Information

**Additional file 1: Table S1.** Primers used for qPCR.

**Additional file 2: Table S2.** *G. hirsutum* fiber transcriptomes assembly statistics.

**Additional file 3: Table S3.** The correlation (R^2^) of gene expression (log_10_ (FPKM+ 1)) between two biological replicates.

**Additional file 4: Table S4.** FPKMs of 47,095 mRNAs in cotton fibers under NI and WD.

**Additional file 5: Table S5.** FPKMs of 13,051 lncRNAs in cotton fibers under NI and WD.

**Additional file 6: Table S6.** List of DEGs in cotton fibers between NI and WD.

**Additional file 7: Table S7.** List of DERs in cotton fibers between NI and WD.

**Additional file 8: Table S8.** List of DEGs potentially regulated by DERs.

**Additional file 9: Table S9.** GO enrichment analysis of DEGs-R.

## Data Availability

The data generated or analyzed during the current study are included in this published article and its supplemental data files. The RNA-seq data are available in the Genome Sequence Archive (https://ngdc.cncb.ac.cn; accession number: CRA005441) or from the corresponding author on reasonable request.

## Abbreviations

ABA: Abscisic acid
DEGs: Differentially expressed genes
DEGs-R: DEGs regulated by DERs
DERs: Differentially expressed lncRNAs
DPA: Days post anthesis
FDT: Fiber developmental transition
FE: Fiber elongation
FI: Fiber initiation
FPKM: Fragments per kilobase per million mapped reads
lincRNAs: Long intergenic noncoding RNAs
lncNATs: Long noncoding natural antisense transcripts
lncRNAs: Long noncoding RNAs
LP: Lint percentage
NI: Normal irrigation
qPCR: Real-time quantitative PCR
RNAi: RNA interference
SCWB: Secondary cell wall biosynthesis
SI: Seed index
SIF: Supplementary irrigation just before flowering
SRWC: Soil relative water content
YRB: The Yellow River Basin
WD: Water deficit
