# Supplementary material for: Novel insights into water-deficit-responsive mRNAs and lncRNAs during fiber development in Gossypium hirsutum
Source: BMC Plant Biol. 2022 Jan 3;22:6. doi: 10.1186/s12870-021-03382-y (PMC8722198; doi:10.1186/s12870-021-03382-y)
Supplement: Supplementary file 1 — Additional file 1: Table S1. Primers used for qPCR. [file 12870_2021_3382_MOESM1_ESM.docx]

| **Table S1** Primers used for qPCR | |
| --- | --- |
| **Primers** | **Primer sequences (5'→3')** |
| LNC_012662-F | GAGCTTTGAAGGGAGCAGTCATC |
| LNC_012662-R | CGCATCCAAAATGAACTGTTACAC |
| LNC_002723-F | CGTGTCGGAGCAGCTACCA |
| LNC_002723-R | CGCAAATGCCACCTTGAATG |
| LNC_008209-F | AATTGCATCTTAACCCTAAAGTTTCCT |
| LNC_008209-R | GCTGCCAGAAGGACCATATTGC |
| LNC_007975-F | ACTCAGCTCAAGGAGGGATGAAG |
| LNC_007975-R | ACAATCATTCAGCGAATTGGTAGAC |
| LNC_005274-F | AAATCCCTGTCTACGGTACCCG |
| LNC_005274-R | CGCTCAAACTACCCACCTAATCTC |
| LNC_011121-F | CCATGTCTACCGCTCAATCTTGT |
| LNC_011121-R | GGTGAGACCCAATTCTAGCAGG |
| LNC_009310-F | TTCCCATCCCTTGAATAGAGCCT |
| LNC_009310-R | GAAAGCCTTGAGATTCCATATCCTG |
| LNC_005312-F | GCAGTCATGGCAGCAAGCA |
| LNC_005312-R | GGACCCAAGAACAGGCAAGG |
| LNC_012164-F | AGCCCCGTGCTCCTAATGTAGAG |
| LNC_012164-R | GCTGGTTGGGTAGCGTAGTAAGG |
| LNC_011638-F | TGGCTGTGGAGAAGTGGACG |
| LNC_011638-R | TGAAGGATTAGGAGTGGGAACATAC |
| Gh_D01G0047-F | ATGTCATCGAATTCAATGTCTGCT |
| Gh_D01G0047-R | CGACTCGATGTGTCTAACATCTTCC |
| Gh_D05G1621-F | GTGGATAAGGAAGACCCAGAGGAG |
| Gh_D05G1621-R | CTTGCTGCCGAAATACTAACCAAC |
| Gh_A05G0770-F | CCCTTTCTTCTTCACTCACCTCAC |
| Gh_A05G0770-R | CCTGTCATCTTCAACCTCCACTTT |
| Gh_D08G2730-F | TGTCTTTGCTGCTCCCCTT |
| Gh_D08G2730-R | TTTGGTGTTGCTATGTAGAAATCTT |
| Gh_A11G2903-F | CGGTGATCGGAGATGAAGGAC |
| Gh_A11G2903-R | AATCACAATACTCCCACTCTGCG |
| Gh_A09G1977-F | TGCCCCGGACAATGTTAAGG |
| Gh_A09G1977-R | GATCATCTCTTCCAAGGTGGTAAT |
| Gh_Sca115726G01-F | AAGCCGATTACTTCGTTCCTCC |
| Gh_Sca115726G01-R | TTCGCACAGTTTTCATCAGCA |
| Gh_D04G0942-F | ATTCTGAACACGCACGCCATC |
| Gh_D04G0942-R | CTCATCTATCACCGTATCCTCGAAG |
| Gh_D08G1970-F | CCACCAGCAGCACCCTTCTT |
| Gh_D08G1970-R | GCCTTGAATACCAACGGTTGC |
| Gh_D03G1452-F | ATGCTGCCTTCGTTTCCCG |
| Gh_D03G1452-R | GGGATCAGCGGCACACAT |
| GhUBQ7-F | GAAGGCATTCCACCTGACCAAC |
| GhUBQ7-R | CTTGACCTTCTTCTTCTTGTGCTTG |
