# Supplementary material for: Novel insights into water-deficit-responsive mRNAs and lncRNAs during fiber development in Gossypium hirsutum
Source: BMC Plant Biol. 2022 Jan 3;22:6. doi: 10.1186/s12870-021-03382-y (PMC8722198; doi:10.1186/s12870-021-03382-y)
Supplement: Supplementary file 2 — Additional file 2: Table S2. G. hirsutum fiber transcriptomes assembly statistics. [file 12870_2021_3382_MOESM2_ESM.docx]

**Table S2** *G. hirsutum* fiber transcriptomes assembly statistics

| **Treatments** | **DPA** | **Replicates** | **Raw reads** | **Clean reads** | **Mapped reads** | **Mapped ratio (%)** |
| --- | --- | --- | --- | --- | --- | --- |
| **NI** | 0 | I | 106 867 564 | 105 893 172 | 93 781 833 | 88.56 |
|  |  | II | 104 945 390 | 103 995 028 | 92 180 242 | 88.64 |
|  | 5 | I | 87 462 858 | 86 605 174 | 76 360 939 | 88.17 |
|  |  | II | 103 971 590 | 103 075 134 | 91 040 538 | 88.32 |
|  | 10 | I | 108 298 622 | 107 398 380 | 93 349 368 | 86.92 |
|  |  | II | 106 603 604 | 105 423 376 | 92 139 657 | 87.40 |
|  | 15 | I | 113 752 938 | 112 294 666 | 96 884 347 | 86.28 |
|  |  | II | 98 504 046 | 97 335 746 | 84 989 788 | 87.32 |
|  | 20 | I | 89 168 236 | 88 565 142 | 75 774 854 | 85.56 |
|  |  | II | 116 623 072 | 115 934 720 | 99 440 388 | 85.77 |
|  | 25 | I | 104 986 402 | 103 654 090 | 86 581 074 | 83.53 |
|  |  | II | 110 698 506 | 109 769 700 | 94 049 562 | 85.68 |
|  | 30 | I | 106 177 316 | 105 331 752 | 90 492 629 | 85.91 |
|  |  | II | 102 023 788 | 100 755 136 | 84 882 328 | 84.25 |
|  | 35 | I | 102 570 636 | 101 394 502 | 81 844 276 | 80.72 |
|  |  | II | 104 147 212 | 103 068 878 | 84 739 702 | 82.22 |
| **WD** | 0 | I | 107 909 942 | 106 733 480 | 92 142 481 | 86.33 |
|  |  | II | 109 424 478 | 108 446 332 | 96 038 684 | 88.56 |
|  | 5 | I | 106 426 870 | 105 544 302 | 93 709 759 | 88.79 |
|  |  | II | 109 714 744 | 108 378 090 | 96 752 036 | 89.27 |
|  | 10 | I | 88 309 192 | 86 671 846 | 75 432 161 | 87.03 |
|  |  | II | 89 151 802 | 87 597 610 | 75 933 518 | 86.68 |
|  | 15 | I | 111 205 974 | 109 438 564 | 95 086 002 | 86.89 |
|  |  | II | 107 282 564 | 105 626 576 | 91 582 341 | 86.70 |
|  | 20 | I | 104 917 944 | 103 013 086 | 86 989 042 | 84.44 |
|  |  | II | 95 535 088 | 94 238 168 | 82 078 384 | 87.10 |
|  | 25 | I | 101 554 774 | 99 615 378 | 86 215 292 | 86.55 |
|  |  | II | 91 032 026 | 88 557 434 | 73 777 807 | 83.31 |
|  | 30 | I | 84 828 040 | 83 166 018 | 69 911 022 | 84.06 |
|  |  | II | 88 992 242 | 87 506 044 | 75 040 690 | 85.75 |
|  | 35 | I | 91 853 422 | 90 280 702 | 76 734 410 | 85.00 |
|  |  | II | 103 194 764 | 101 081 552 | 83 562 880 | 82.67 |

Note: DPA, days post anthesis.
