# Supplementary material for: Novel insights into water-deficit-responsive mRNAs and lncRNAs during fiber development in Gossypium hirsutum
Source: BMC Plant Biol. 2022 Jan 3;22:6. doi: 10.1186/s12870-021-03382-y (PMC8722198; doi:10.1186/s12870-021-03382-y)
Supplement: Supplementary file 3 — Additional file 3: Table S3. The correlation (R2) of gene expression (log10 (FPKM+ 1)) between two biological replicates. [file 12870_2021_3382_MOESM3_ESM.docx]

**Table S3** The correlation (R^2^) of gene expression (log_10_ (FPKM+1)) between two biological replicates

|  | 0 DPA | 5 DPA | 10 DPA | 15 DPA | 20 DPA | 25 DPA | 30 DPA | 35 DPA |
| --- | --- | --- | --- | --- | --- | --- | --- | --- |
| NI | 0.90 | 0.93 | 0.94 | 0.94 | 0.94 | 0.94 | 0.92 | 0.92 |
| WD | 0.93 | 0.94 | 0.94 | 0.94 | 0.92 | 0.87 | 0.94 | 0.94 |

Note: DPA, days post anthesis.
